# Diagnoses of Mental Health Disorders Among U.S. Active Component Service Members, 2021–2025

**Published:** 2026-08-20

## Abstract

Safeguarding the mental well-being of military service members is a critical strategic imperative for force readiness. This report summarizes the incidence of mental health disorders among U.S. active component service members (ACSMs) from January 1, 2021 through December 31, 2025. During the surveillance period, 582,279 ACSMs received at least 1 mental health diagnosis, and 1,050,231 total incident diagnoses were made. Overall incidence rates peaked in 2023 before declining slightly, through 2025. Adjustment, anxiety, and depressive disorders comprise the clinical burden majority of mental health care, accounting for 66.5% of all incident diagnoses. Diagnostic comorbidity was notably high, with 48.4% of affected ACSMs receiving diagnoses in multiple mental health categories. Female ACSMs generally experienced higher rates for most mental health conditions, while male ACSMs had higher incidence rates for alcohol- and substance-related disorders. Vulnerabilities related to occupation are prominent: Health care personnel evinced highest rates for most disorders, while combat-related functions were most closely associated with substance-related conditions. These findings underscore the ongoing need to prioritize access to mental health services for ACSMs. The high volume of co-occurring diagnoses and distinct occupational risks require robust, multi-disciplinary clinical resources to sustain a resilient and ready joint force.

**What are the new findings?**

From 2021 through 2025, over 1 million incident mental health diagnoses for U.S. active component service members occurred, with most of that health care burden a result of disorders related to adjustment, anxiety, and depression. Notably, those three disorder categories accounted for approximately two-thirds (66.5%) of all incident mental health diagnoses during the surveillance period.

**What is the impact on readiness and force health protection?**

The sustained high incidence of mental health disorders (10,990.6 per 100,000 person-years) in 2025 and high rates of diagnostic comorbidity among active component service members directly compromise operational readiness. Variations in diagnosis rates by sex, service branch, occupation, and length of service underscore the need for targeted, multidisciplinary interventions.

A modern, effective fighting force requires service members who are not only physically fit but also cognitively and psychologically resilient. ^1^ The complex nature of global security environments, coupled with the rigorous demands of continuous operations, necessitates a total force capable of adapting and performing under extreme pressure. ^2^ Safeguarding the mental well-being of service members is not merely a medical objective, but a critical strategic imperative essential for sustained mission success and national security. ^3^

In 2025, mental health disorders were a primary factor of health care provision among U.S. active component service members (ACSMs), accounting for a significant portion of both ambulatory care and hospitalizations. ^4^ These disorders led to more hospital bed days than any other condition, contributing to more than half (51.0%) of all hospital bed days for this population. ^4^ Although injuries were the primary factor in ambulatory health care encounters, mental health disorders constituted the second-largest category of total medical visits, affecting nearly 275,000 service members and generating approximately 2.6 million clinical encounters. ^4^ Conditions such as adjustment disorders, major depressive disorders, and alcohol dependence require substantial health care resources, evidenced by the prolonged hospital stays associated with them, which frequently exceeded 10 days. ^5^

To address this substantial clinical burden, the Department of War (DOW) has increasingly prioritized policies designed to destigmatize behavioral health care, protect patient privacy, and encourage early intervention. Central to this effort is implementation of the Brandon Act, ^6^ which empowers service members to independently request and receive expedited mental health evaluations through their chains of command. Department Instruction 6490.08 reinforces crucial privacy protections for service members by strictly limiting notifications to commands within critical safety or mission impact parameters, thereby mitigating fear of career repercussions. ^7^

Understanding the epidemiology of these mental health conditions is vital to optimize medical resource allocation and develop targeted prevention strategies. This report presents a summary of the numbers, types, and incidence rates (IRs) of mental health disorder diagnoses among U.S. ACSMs during a 5-year surveillance period, from 2021 through 2025.

## Methods

The surveillance population for this study included all individuals who served in the active components of the U.S. Army, Navy, Air Force, Marine Corps, Coast Guard, and Space Force at any time during the surveillance period, January 1, 2021 through December 31, 2025. Because only 2023 Space Force personnel data were available, Space Force members were combined with Air Force personnel for this analysis.

All data utilized to determine mental health diagnoses were derived from records routinely maintained in the Defense Medical Surveillance System (DMSS). DMSS records document both ambulatory health care encounters and hospitalizations of U.S. active component service members at permanent military and civilian (if reimbursed through the Military Health System, or MHS) hospitals and clinics. Diagnoses were also derived from records of medical encounters of deployed service members documented in the Theater Medical Data Store (TMDS) within DMSS.

Mental health disorder diagnoses were ascertained from records of medical encounters that included International Classification of Diseases, 9th and 10th revisions (ICD-9/ICD-10) mental health disorder-specific diagnosis codes (ICD-9: 290–319; ICD-10: F01–F99) **(Table 1)** in the first or second diagnostic position. Although the MHS transitioned to ICD-10 coding on October 1, 2015, ICD-9 codes were included in this analysis, as some TMDS encounters still contain ICD-9 diagnoses necessary to identify and exclude prevalent cases documented in records before October 1, 2015. Diagnoses of pervasive developmental disorder (ICD-9: 299.*; ICD-10: F84.*), specific delays in development (ICD-9: 315.*; ICD-10: F80.*–F82.*, F88–F89), mental retardation (ICD-9: 317.*–319.*; ICD-10: F70–F79), tobacco use disorder and nicotine dependence (ICD-9: 305.1; ICD-10: F17.*), and post-concussion syndrome (ICD-9: 310.2; ICD-10: F07.81) were excluded from analysis.

**TABLE 1.** Mental Health Disorder Classifications

Category	ICD-9 Codes	ICD-10 Codes
Acute stress disorder	308 *	F43.0
Adjustment disorders	309, 309.0, 309.1, 309.2, 309.21, 309.22, 309.23, 309.24, 309.28, 309.29, 309.3, 309.4, 309.8, 309.82, 309.83, 309.89, 309.9	F43.2 * , F43.8 * F43.9, F93.0, F94.8, F94.9
Alcohol-related disorder	291.0, 291.81, 303.9, 303.90, 303.91, 303.92, 303.93, 303.00, 303.01, 303.02, 303.03, 305.00, 305.01, 305.02, 305.03	F101 * , F102 *
Substance-related disorder	304 * , 305.2 * –305.9 *	F111 * , F112 * , F121 * , F122 * , F131 * , F132 * , F141 * , F142 * , F151 * , F152 * , F161 * , F162 * , F181 * , F182 * , F191 * , F192 *
Anxiety disorders	300.0 * , 300.2 * , 300.3	F40 * , F41 * , F42 *
PTSD	309.81	F431 *
Depressive disorder	296.2, 296.20, 296.21, 296.22, 296.23, 296.24, 296.25, 296.26, 296.3, 296.30, 296.31, 296.32, 296.33, 296.35, 296.36, 296.82, 296.34, 296.9, 296.90, 296.99, 300.4, 311	F32 * , F33 * , F34, F341, F348, F349, F348.1, F348.9, F39
Eating disorders	307.1, 307.50, 307.51, 307.59	F50.0 * , F50.2 * , F50.8 * , F50.9
Factitious disorders	300.16, 300.19, 301.51	F681 *
Bipolar disorder	296.0, 296.00, 296.01, 296.02, 296.03, 296.04, 296.05, 296.06, 296.1, 296.10, 296.11, 296.12, 296.13, 296.14, 296.15, 296.16, 296.4, 296.40, 296.41, 296.42, 296.43, 296.44, 296.45, 296.46, 296.5, 296.50, 296.51, 296.52, 296.53, 296.54, 296.55, 296.56, 296.6, 296.60, 296.61, 296.62, 296.63, 296.64, 296.65, 296.66, 296.7, 296.8, 296.80, 296.81, 296.89, 301.13	F30 * , F31 * , F340
Personality disorders	301, 301.0, 301.1, 301.10, 301.11, 301.12, 301.2, 301.20, 301.21, 301.22, 301.3, 301.4, 301.5, 301.50, 301.59, 301.6, 301.7, 301.8, 301.81, 301.82, 301.83, 301.84, 301.89, 301.9	F21, F60 *
Schizophrenia	295 *	F20 * , F25 *
Psychotic disorders (other psychoses)	293.81, 293.82, 297.0, 297.1, 297.2, 297.3, 297.8, 297.9, 298.0, 298.1, 298.2, 298.3, 298.4, 298.8, 298.9	F06.0, F06.2, F22, F23, F24, F28, F29
Other mental health diagnoses	Any other not-excluded code between 290–319	Any other not-excluded code between F01–F99
Exclusions	305.1, 310.2, 299 * , 315 * , 317 * –319 *	F17 * , F0781, F70–F79, F80 * , F81 * , F82 * , F84 * , F88–F89

Abbreviations: ICD-9, International Classification of Diseases, 9th Revision; ICD-10, International Classification of Diseases, 10th Revision; PTSD, post-traumatic stress disorder.

* Asterisk (*) indicates that any subsequent digit, character included.

Each incident diagnosis of a mental health disorder was defined using the corresponding Armed Forces Health Surveillance Case Definition. ^8^ For most mental health disorders, a case was defined by either a hospitalization with an indicator diagnosis in the first or second diagnostic position; 2 outpatient or TMDS visits within 180 days documented with indicator diagnoses (from the same mental health disorder category) in the first or second diagnostic position; or a single outpatient visit in a psychiatric or mental health care specialty setting (defined by Medical Expense and Performance Reporting System [MEPRS] code beginning with ‘BF’) with an indicator diagnosis in the first or second diagnostic position.

The surveillance case definitions for schizophrenia, acute stress disorder, and eating disorders included some exceptions to the case parameters described. The case definition for schizophrenia required either a single hospitalization with a diagnosis of schizophrenia in the first or second diagnostic position or 4 outpatient or TMDS encounters with a diagnosis of schizophrenia in the first or second diagnostic position. Schizophrenia cases who remained in the military for more than 2 years after becoming incident cases were excluded, as those cases were assumed to have been misdiagnosed. The case definition for acute stress disorders required 1 encounter with an indicator diagnosis in any diagnostic position, due to its transient diagnosis. Eating disorder cases required 1 inpatient encounter with an indicator diagnosis in the first or second diagnostic position, or a single outpatient or TMDS encounter with an indicator diagnosis in the primary diagnostic position.

Service members diagnosed with 1 or more mental health disorders before the surveillance period (i.e., prevalent cases) were not considered at risk of incident diagnoses of the same conditions during the period. Service members diagnosed with more than 1 mental health disorder during the surveillance period were considered incident cases in each category in which they fulfilled the case-defining criteria. Service members could be considered incident cases only once in each mental health disorder-specific category.

## Results

### Numbers and incidence rates of mental health diagnoses

During the 5-year surveillance period, 582,279 ACSMs were diagnosed with at least 1 mental health disorder; of those individuals, 282,073 (48.4%) were diagnosed with mental health disorders in more than 1 diagnostic category **(Table 2)** . Overall, 1,050,231 incident diagnoses of mental health disorders were recorded in all diagnostic categories. Annual IRs of at least 1 mental health disorder increased from 10,254.3 per 100,000 person-years (p-yrs) in 2021 to 11,679.9 per 100,000 p-yrs in 2023, then decreased slightly to 11,539.6 in 2024 and 10,990.6 per 100,000 p-yrs in 2025 **(Table 2)** .

**TABLE 2.** Incident Diagnoses and Rates of Mental Health Disorders, Active Component, U.S. Armed Forces, 2021–2025

Characteristics	Total, 2021–2025		2021		2022		2023		2024		2025	
	No.	Rate ^b^	No.	Rate ^b^	No.	Rate ^b^	No.	Rate ^b^	No.	Rate ^b^	No.	Rate ^b^
**Category of mental health disorder** ^a^												
Adjustment disorders	286,375	5,122.0	61,149	5,203.0	62,208	5,514.0	56,059	5,118.0	52,976	4,904.0	53,983	4,855.0
Anxiety	225,557	3,814.0	37,654	3,008.0	45,348	3,768.0	48,681	4,190.0	48,312	4,254.0	45,562	3,922.0
Depression	186,257	3,091.0	34,451	2,728.0	39,118	3,209.0	39,441	3,332.0	37,659	3,235.0	35,588	2,974.0
Other mental health disorder	131,157	2,164.0	24,355	1,926.0	26,746	2,184.0	26,020	2,181.0	26,781	2,281.0	27,255	2,261.0
PTSD	101,969	1,611.0	17,311	1,312.0	20,835	1,630.0	22,386	1,798.0	21,622	1,764.0	19,815	1,573.0
Alcohol-related disorder	69,779	1,108.0	14,247	1,092.0	14,885	1,174.0	13,590	1,096.0	13,322	1,089.0	13,735	1,091.0
Personality disorders	15,585	239.0	3,296	244.0	3,586	273.0	3,193	249.0	2,838	224.0	2,672	205.0
Substance-related disorder	14,670	225.0	3,361	249.0	3,489	266.0	2,920	227.0	2,543	200.0	2,357	181.0
Bipolar disorder	8,491	130.0	1,833	135.0	1,960	149.0	1,806	140.0	1,565	123.0	1,327	102.0
Eating disorders	3,911	60.0	708	52.0	885	67.0	808	63.0	779	61.0	731	56.0
Other psychoses	3,742	57.0	844	62.0	820	62.0	765	59.0	674	53.0	639	49.0
Schizophrenia	1,464	22.0	310	23.0	309	23.0	300	23.0	282	22.0	263	20.0
Acute stress disorder	1,181	18.0	262	19.0	291	22.0	221	17.0	214	17.0	193	15.0
Factitious disorders	93	1.0	16	1.0	24	2.0	18	1.0	21	2.0	14	1.0
Total	1,050,231		199,797		220,504		216,208		209,588		204,134	
** Individuals, *n***												
More than 1 type of diagnosis ^c^	282,073	4,306.4	43,455	3,203.6	48,865	3,703.4	47,854	3,706.3	45,948	3,607.9	44,075	3,365.7
Any diagnosis ^d^	582,279	8,889.6	139,094	10,254.3	152,608	11,566.0	150,807	11,679.9	146,961	11,539.6	143,924	10,990.6

Abbreviations: No., number; PTSD, post-traumatic stress disorder; *n* , number.

a An individual could be a case within a category only once per lifetime, i.e., censored person-time.

b Rate per 100,000 person-years.

c Rate per 100,000 person-years (individual continually at risk- uncensored person-time).

d Defined as a unique occurrence of any mental health diagnosis.

Over the entire surveillance period, 95% of all incident mental health diagnoses were attributed to adjustment disorders (n=286,375, 27.3%), anxiety disorders (n=225,557, 21.5%), depression disorders (n=186,257, 17.7%), ‘other’ mental health disorders (n=131,157, 12.5%), PTSD (n=101,969, 9.7%), and alcohol-related disorders (n=69,779, 6.6%) **(Table 2)** . A relatively small number of incident diagnoses for personality disorders (n=15,585, 1.5%), substance-related disorders (n=14,670, 1.4%), bipolar disorder (n=8,491, 0.8%), eating disorders (n=3,911, 0.4%), other psychoses (n=3,742, 0.4%), schizophrenia (n=1,464, 0.1%), acute stress disorders (n=1,181, 0.1%), and factitious disorders (n=93, 0.01%) contributed to the incident mental health disorder diagnoses of ACSMs.

Annual IRs for adjustment, personality, substance-related, bipolar, and eating disorders increased from 2021 to 2022, then subsequently decreased. Anxiety evinced a gradual and steady increase over the 4-year surveillance period before declining in 2025. Depression and PTSD began to decrease in 2024 **(Table 2)** .

### Co-occurring mental health diagnoses

Individuals with mental health disorders are often co-diagnosed with other mental health disorders. For example, adjustment disorders were often co-diagnosed with other mental health disorders, ranging from co-diagnoses with 34.6% of substance-related disorder diagnoses to 57.7% of personality disorder diagnoses during the surveillance period. Depressive disorders were also often co-diagnosed with all other mental health disorders, ranging from co-diagnoses with 26.6% of substance-related disorder diagnoses to 58.8% of bipolar disorder diagnoses. Additionally, co-occurring anxiety disorders were highly prevalent among individuals with other primary diagnoses, affecting 50.5% of those with factitious disorders, 48.0% of those with bipolar disorders, 47.8% of those with depressive disorders, 44.4% of those with eating disorders, 42.8% of those with PTSD, 41.8% of those with personality disorders, and 37.5% of those with acute stress disorder **(Table 3)** .

**TABLE 3.** Co-Morbid Incident Mental Health Disorder Diagnoses ^a^ , Active Component, U.S. Armed Forces, 2021–2025

Categories of Mental Health Disorders	Adjustment Disorders		Alcohol-related Disorder		Substance-related Disorder		Anxiety		PTSD		Depression		Bipolar Disorder	
	No.	%	No.	%	No.	%	No.	%	No.	%	No.	%	No.	%
Adjustment disorders	286,375	—	25,596	36.7	5,071	34.6	92,666	41.1	44,399	43.5	88,020	47.3	3,666	43.2
Alcohol-related disorder	25,596	8.9	69,779	—	7,010	47.8	18,169	8.1	9,702	9.5	21,381	11.5	1,486	17.5
Substance-related disorder	5,071	1.8	7,010	10.1	14,670	—	3,099	1.4	1,462	1.4	3,898	2.1	506	6.0
Anxiety	92,666	32.4	18,169	26.0	3,099	21.1	225,557	—	43,619	42.8	88,999	47.8	4,074	48.0
PTSD	44,399	15.5	9,702	13.9	1,462	10.0	43,619	19.3	101,969	—	40,879	22.0	2,611	30.8
Depression	88,020	30.7	21,381	30.6	3,898	26.6	88,999	39.5	40,879	40.1	186,257	—	4,989	58.8
Bipolar disorder	3,666	1.3	1,486	2.1	506	3.5	4,074	1.8	2,611	2.6	4,989	2.7	8,491	—
Personality disorders	8,994	3.1	2,991	4.3	725	4.9	6,520	2.9	3,803	3.7	8,276	4.4	1,283	15.1
Schizophrenia	579	0.2	241	0.4	136	0.9	505	0.2	256	0.3	746	0.4	290	3.4
Other psychoses	1,656	0.6	661	1.0	426	2.9	1,274	0.6	641	0.6	1,789	1.0	706	8.3
Acute stress disorder	501	0.2	101	0.1	22	0.2	443	0.2	302	0.3	388	0.2	39	0.5
Eating disorders	1,656	0.6	416	0.6	66	0.5	1,736	0.8	1,045	1.0	1,808	1.0	170	2.0
Factitious disorders	56	0.0	9	0.0	4	0.0	47	0.0	15	0.0	49	0.0	4	0.1
Other mental health disorder	46,258	16.2	19,827	28.4	5,644	38.5	39,719	17.6	19,325	19.0	34,865	18.7	2,058	24.2
Total	286,375		69,779		14,670		225,557		101,969		186,257		8,491	

	Personality Disorders		Schizophrenia		Other Psychoses		Acute Stress		Eating Disorders		Factitious Disorder		Other	
	No.	%	No.	%	No.	%	No.	%	No.	%	No.	%	No.	%
Adjustment disorders	8,994	57.7	579	39.6	1,656	44.3	501	42.4	1,656	42.3	56	60.2	46,258	35.3
Alcohol-related disorder	2,991	19.2	241	16.5	661	17.7	101	8.6	416	10.6	9	9.7	19,827	15.1
Substance-related disorder	725	4.7	136	9.3	426	11.4	22	1.9	66	1.7	4	4.3	5,644	4.3
Anxiety	6,520	41.8	505	34.5	1,274	34.1	443	37.5	1,736	44.4	47	50.5	39,719	30.3
PTSD	3,803	24.4	256	17.5	641	17.1	302	25.6	1,045	26.7	15	16.1	19,325	14.7
Depression	8,276	53.1	746	51.0	1,789	47.8	388	32.9	1,808	46.2	49	52.7	34,865	26.6
Bipolar disorder	1,283	8.2	290	19.8	706	18.9	39	3.3	170	4.4	4	4.3	2,058	1.6
Personality disorders	15,585	—	202	13.8	522	14.0	54	4.6	388	9.9	15	16.1	4,130	3.2
Schizophrenia	202	1.3	1,464	—	923	24.7	5	0.4	13	0.3	5	5.4	461	0.4
Other psychoses	522	3.4	923	63.1	3,742	—	17	1.4	25	0.6	16	17.2	1,179	0.9
Acute stress disorder	54	0.4	5	0.3	17	0.5	1,181	—	11	0.3	0	0.0	351	0.3
Eating disorders	388	2.5	13	0.9	25	0.7	11	0.9	3,911	—	3	3.2	1,269	1.0
Factitious disorders	15	0.1	5	0.3	16	0.4	0	0.0	3	0.1	93	—	35	0.0
Other mental health disorder	4,130	26.5	461	31.5	1,179	31.5	351	29.7	1,269	32.5	35	37.6	131,157	—
Total	15,585		1,464		3,742		1,181		3,911		93		131,157	

Abbreviation: PTSD, post-traumatic stress disorder; No., number.

a Mental health disorder diagnosis at any time during surveillance period.

### Incidence rates of mental health diagnoses by sex

In general, most incident mental health disorder diagnoses were more prevalent among female service members, but alcohol- and substance-related disorders were more prevalent in male service members during the 5-year surveillance period. Schizophrenia was diagnosed at a higher rate in male service members in 2024 and 2025 **(Figures 1a** – **2b)** .

**FIGURE 1a.**
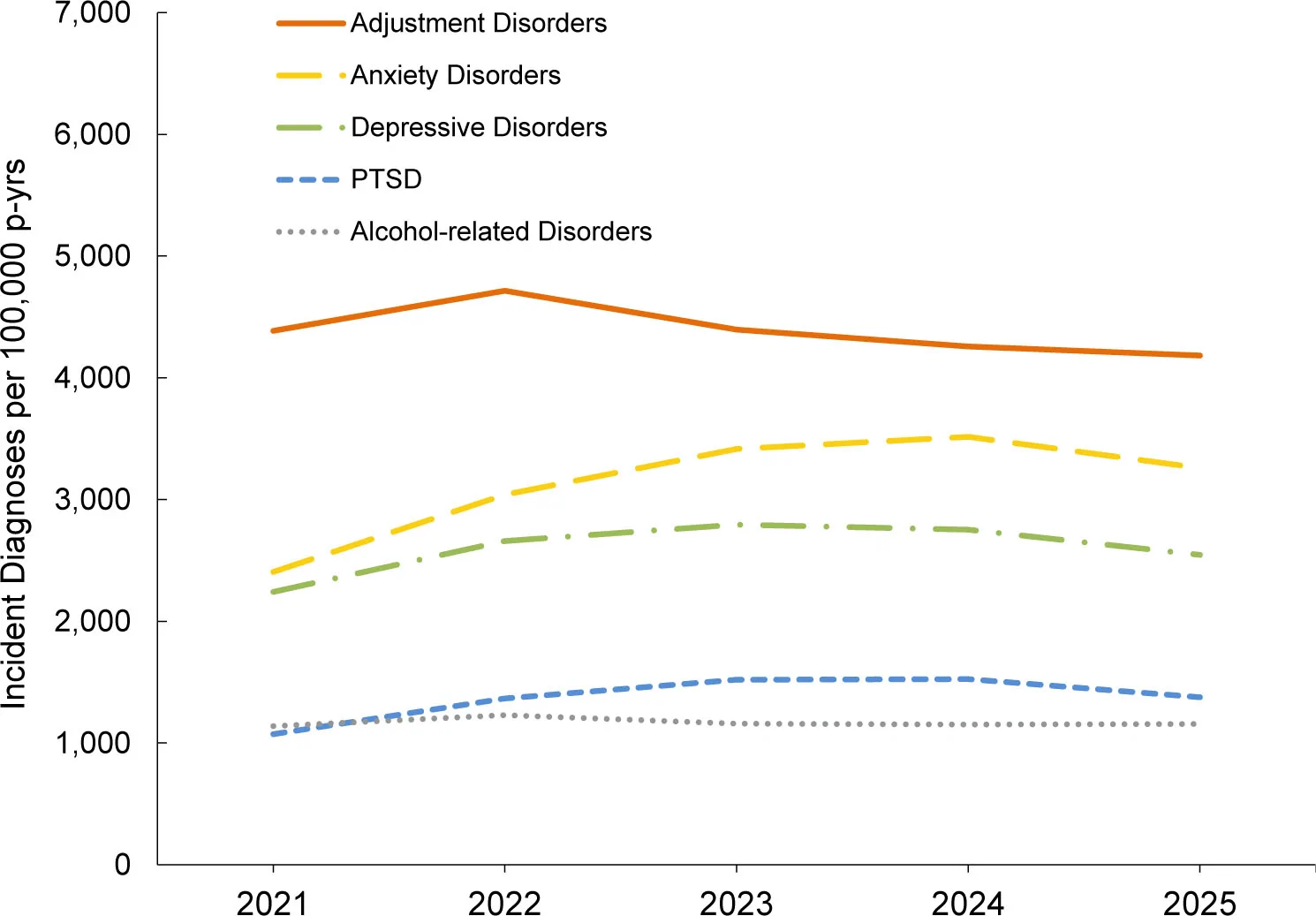
Annual Incidence Rates for the Five Most Frequent Mental Health Disorder Diagnoses, Active Component Service Men, U.S. Armed Forces 2021–2025

**FIGURE 1b.**
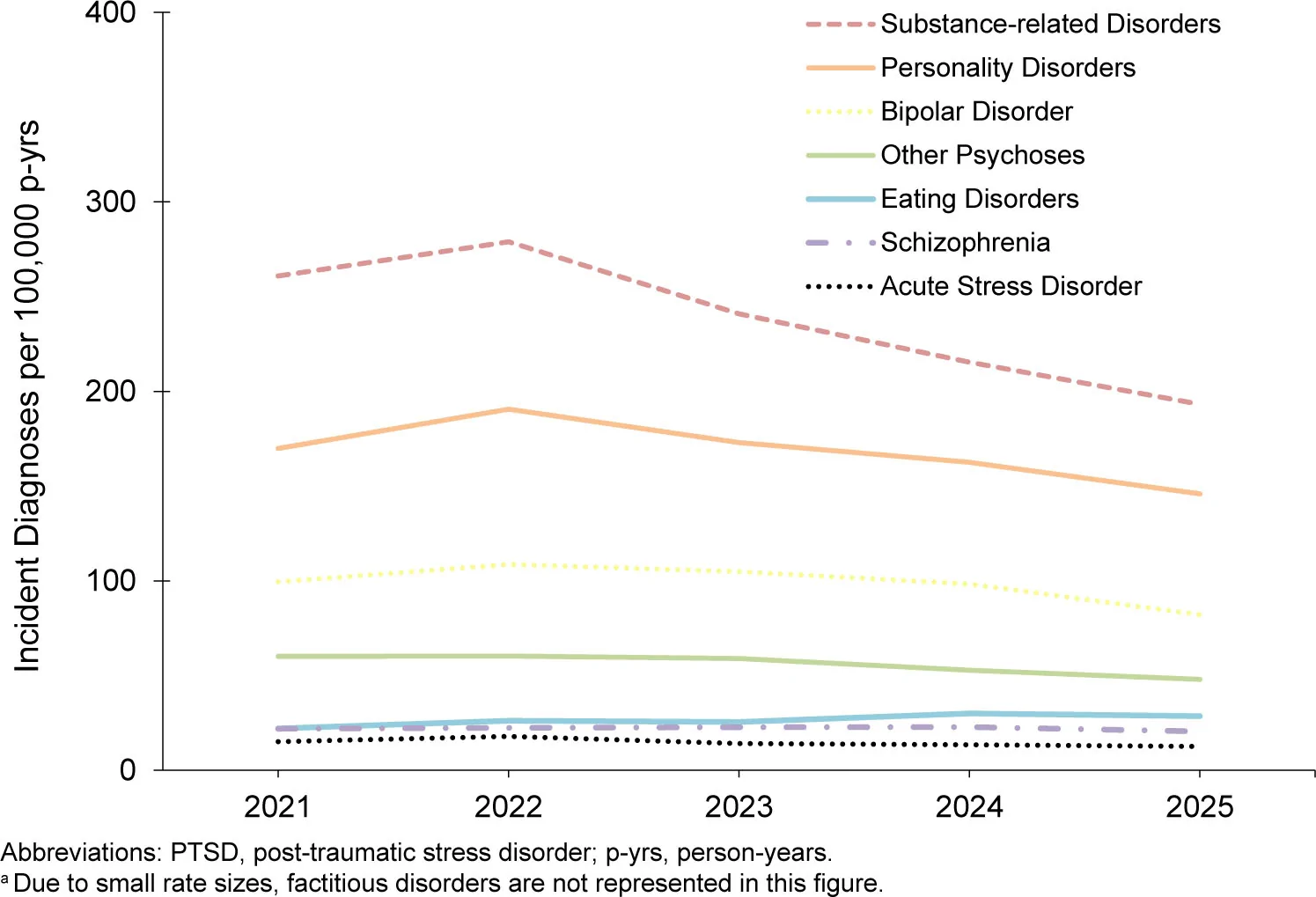
Annual Incidence Rates ^a^ for the Next Most Frequent Mental Health Diagnoses, Active Component Service Men, U.S. Armed Forces 2021–2025

**FIGURE 2a.**
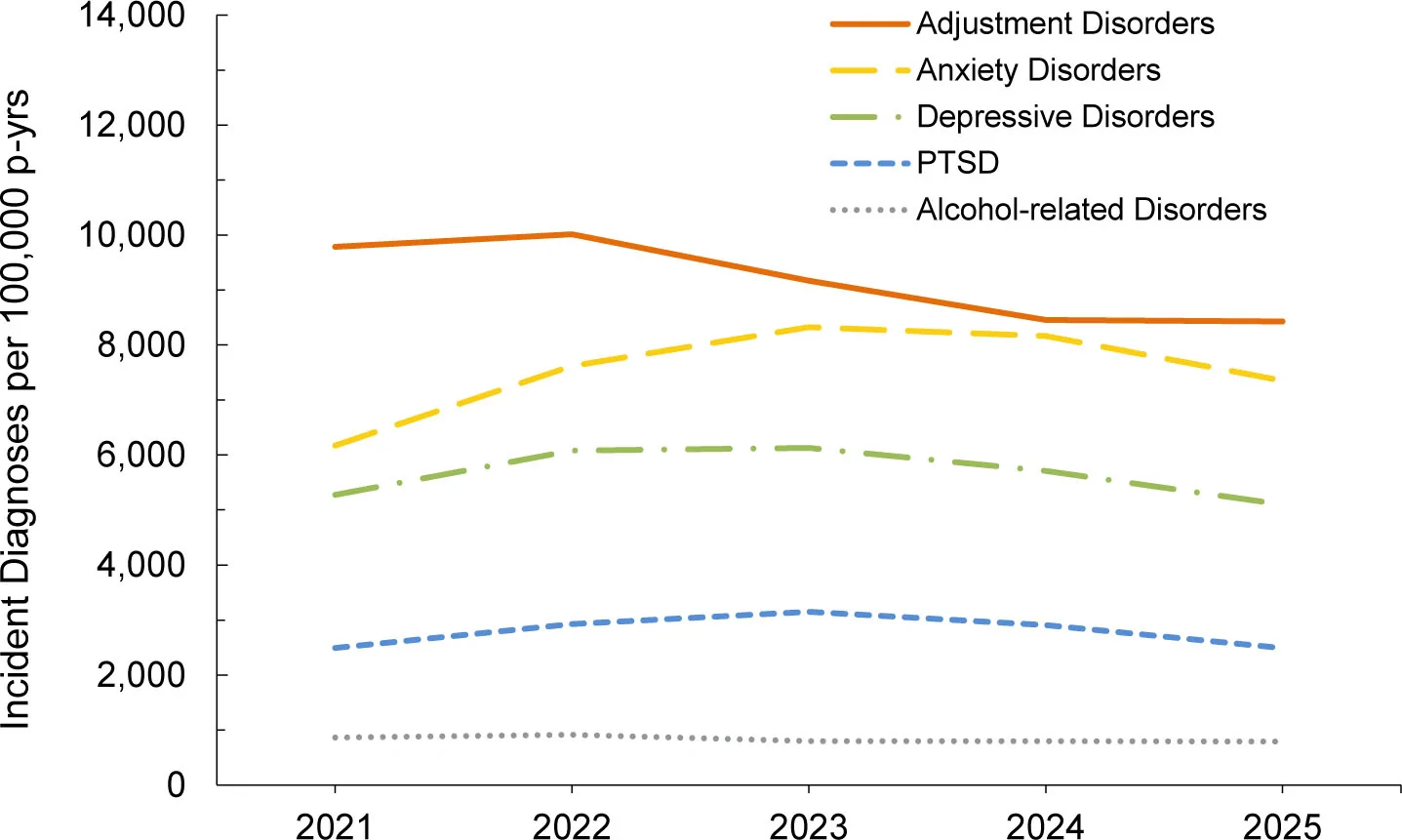
Annual Incidence Rates of Five Most Frequent Mental Health Disorder Diagnoses, Active Component Service Women, U.S. Armed Forces 2021–2025

**FIGURE 2b.**
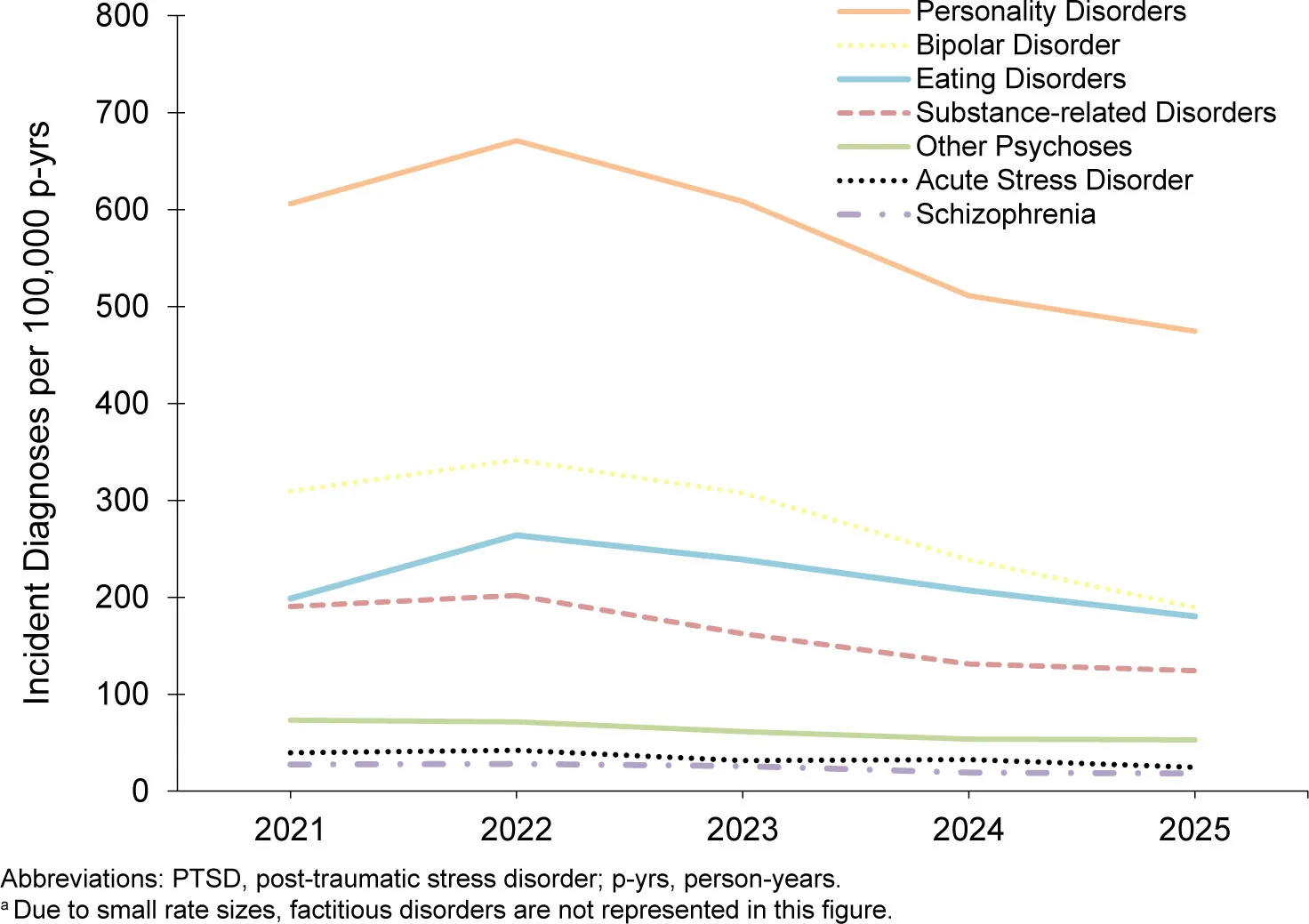
Annual Incidence Rates ^a^ for Next Most Frequent Mental Health Diagnoses, Active Component Service Women, U.S. Armed Forces 2021–2025

Among male service members, rates of diagnoses including adjustment disorders, alcohol-related disorders, substance-related disorders, bipolar disorders, personality disorders, other psychoses, and acute stress disorders decreased in 2023 and continued a downward trend thereafter, while anxiety disorders among male service members increased through 2024 and then decreased in 2025 **(Figures 1a** , **1b)** .

Rates of mental health disorder diagnoses among female service members generally mirrored those of their male counterparts, with the notable exceptions of eating disorders (since 2023) and factitious disorders (since 2024); unlike the fluctuating rates among male service members, those 2 conditions showed a continuous downward trend among female service members. As of 2025, adjustment disorder evinced the highest IRs among women, followed by anxiety disorders, depressive disorders, other mental health disorders, and PTSD. Throughout the 5-year surveillance period, eating disorders occurred 6–10 times more frequently in female service members than in men, and personality disorders were identified 3.1–3.6 times more frequently in women **(Figures 2a** , **2b)** .

### Incidence rates of mental health diagnoses by age

Rates of most mental health disorders varied by age, with adjustment disorders exhibiting the highest incidence among all age groups **(Figure 3)** . Service members younger than age 20 years had the highest IR of other psychoses, compared to all other age groups. Rates of alcohol- and substance-related disorders, along with personality disorders, bipolar disorders, eating disorders, and schizophrenia, were highest for service members ages 20-24 years, then declined with increasing age. As age increased, PTSD increased, while adjustment disorders, anxiety disorders, depressive disorders, and acute stress disorders and factitious disorders evidenced fluctuating trends. ACSMs ages 40-49-years had the highest IRs of adjustment disorders, anxiety disorders, and depressive disorders; and those older than age 50 years had the highest incidence of PTSD and factitious disorders. Rates of adjustment disorders, anxiety disorders, and depressive disorders began increasing among those older than age 30 years until age 49 years, then declining thereafter among those older than age 50 years.

**FIGURE 3.**
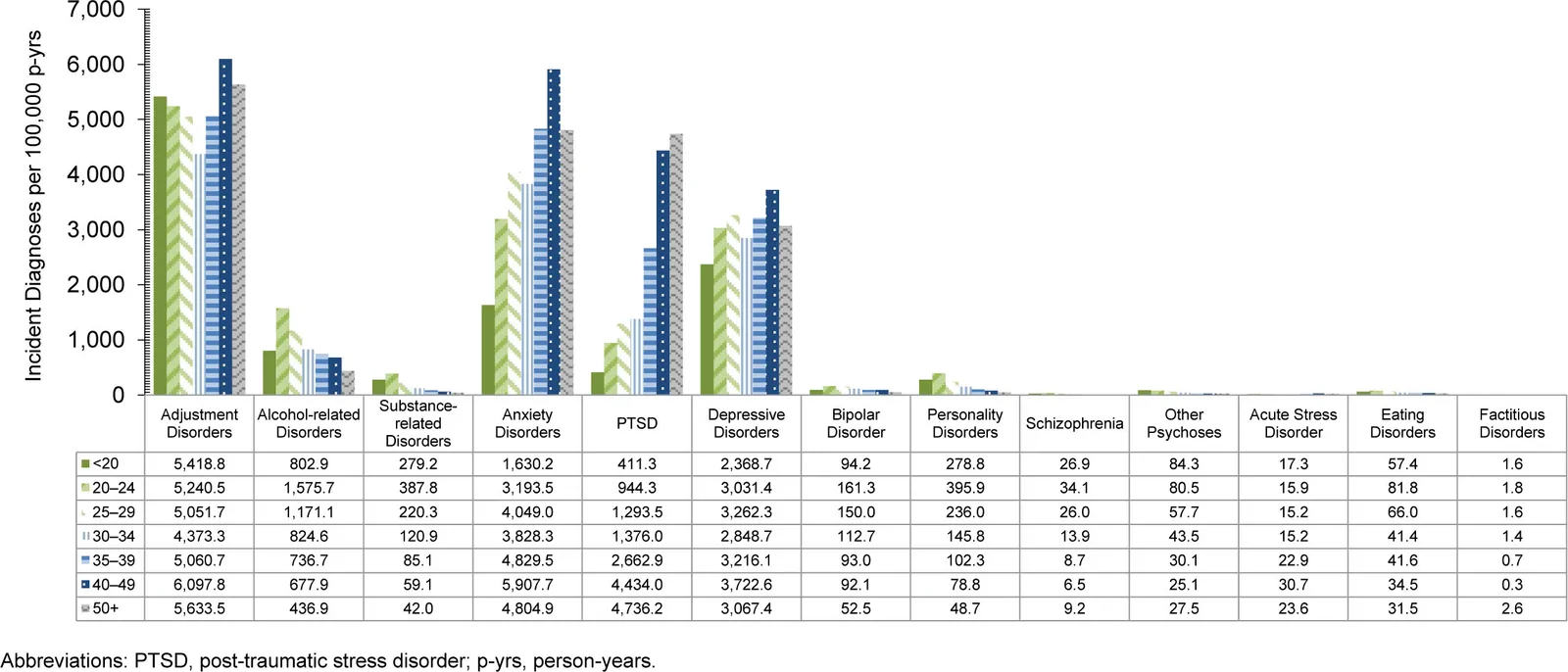
Incidence Rates of Mental Health Disorder Diagnoses by Category and Age Group, Active Component, U.S. Armed Forces, 2021–2025

### Incidence rates of mental health diagnoses by service

The Army evidenced the highest IRs of mental health disorders, notably adjustment disorders, alcohol-related disorders, substance related disorders, PTSD, schizophrenia, other psychoses, eating disorders, and factitious disorders. The Navy accounted for the highest IRs of depressive, personality, and bipolar disorders, while the Coast Guard accounted for the highest IRs of anxiety and acute stress disorders **(Figure 4)** .

**FIGURE 4.**
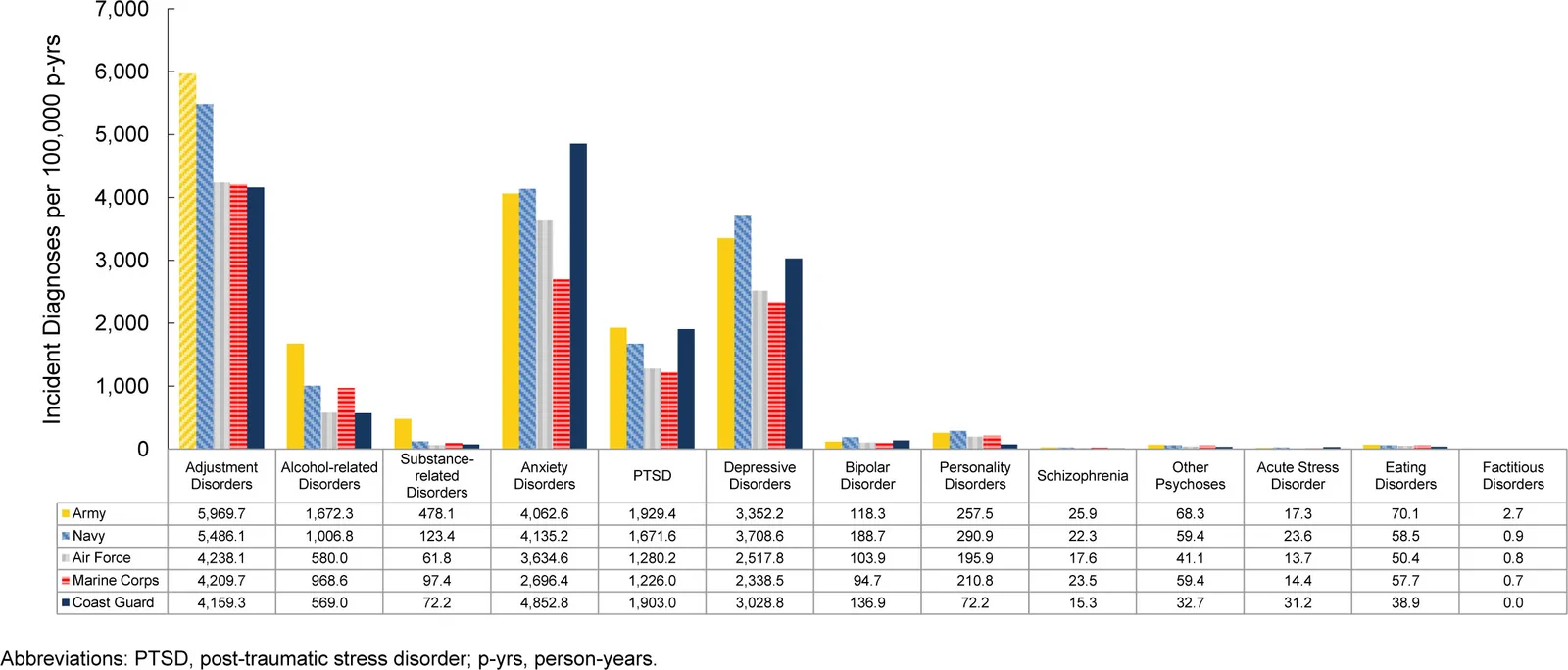
Incidence Rates of Mental Health Disorder Diagnoses by Category and Branch of Service, Active Component, U.S. Armed Forces, 2021–2025

### Incidence rates of mental health diagnoses by occupation

Rates of adjustment disorders, anxiety disorders, depressive disorders, PTSD, personality disorders, bipolar disorders, eating disorders, and acute stress disorders were generally highest in health care occupations. Service members in combat-related roles exhibited the highest IRs of alcohol- and substance-related disorders and factitious disorders, while those in motor transport had the highest rates of other psychoses and schizophrenia. By contrast, pilots and air crew personnel showed the lowest IRs of mental health disorders **(Figure 5)** .

**FIGURE 5.**
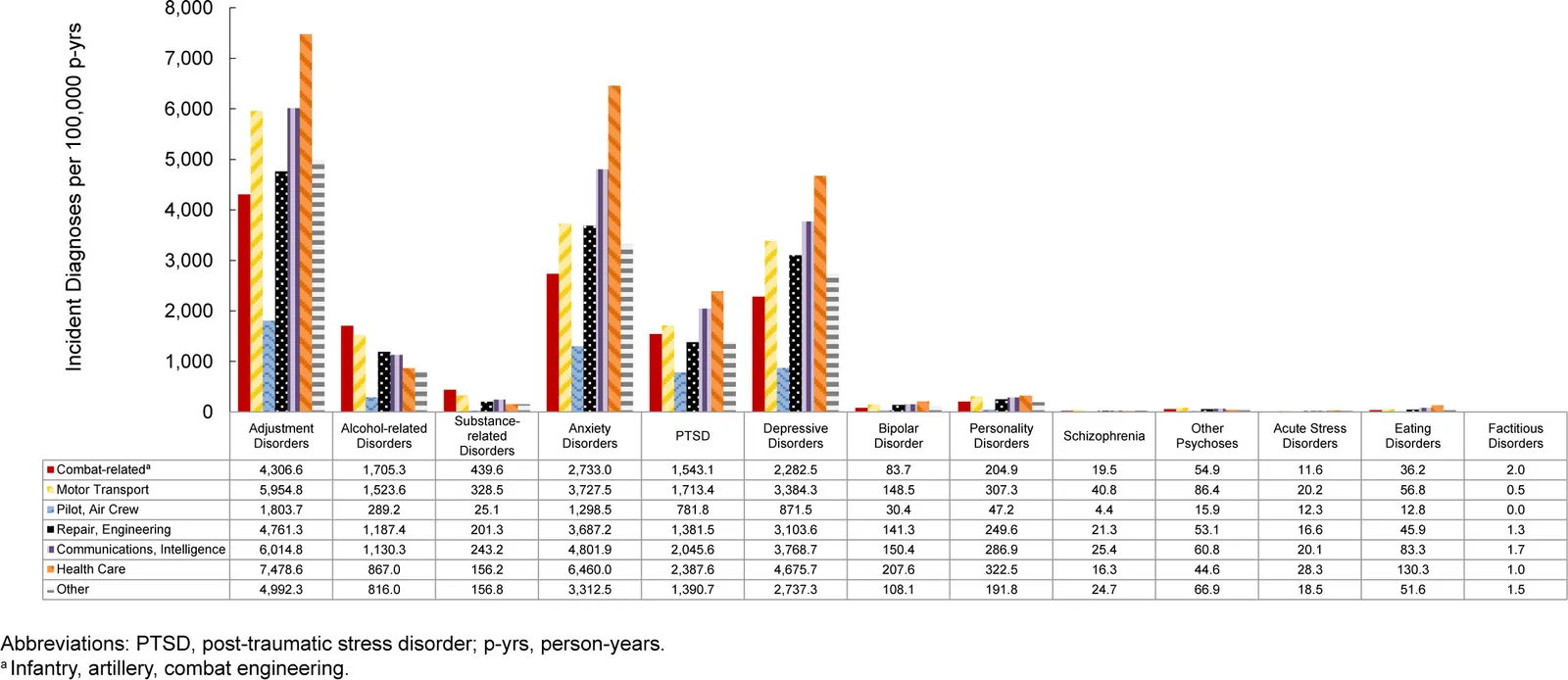
Incidence Rates of Mental Health Disorder Diagnoses by Category and Military Occupation, Active Component, U.S. Armed Forces, 2021–2025

### Incidence rates of mental health diagnoses by time in service

Rates of mental health disorder diagnoses differ by length of service, with highest IRs of other psychoses and acute stress disorder diagnoses occurring among ACSMs with less than 6 months of service. For those who served 12–36 months, the most common diagnoses were adjustment disorders, alcohol-related disorders, substance-related disorders, personality disorders, bipolar disorders, eating disorders, and schizophrenia. Among those who served 36 months or longer, anxiety disorders, depressive disorders, and PTSD were most common **(Figure 6)** .

**FIGURE 6.**
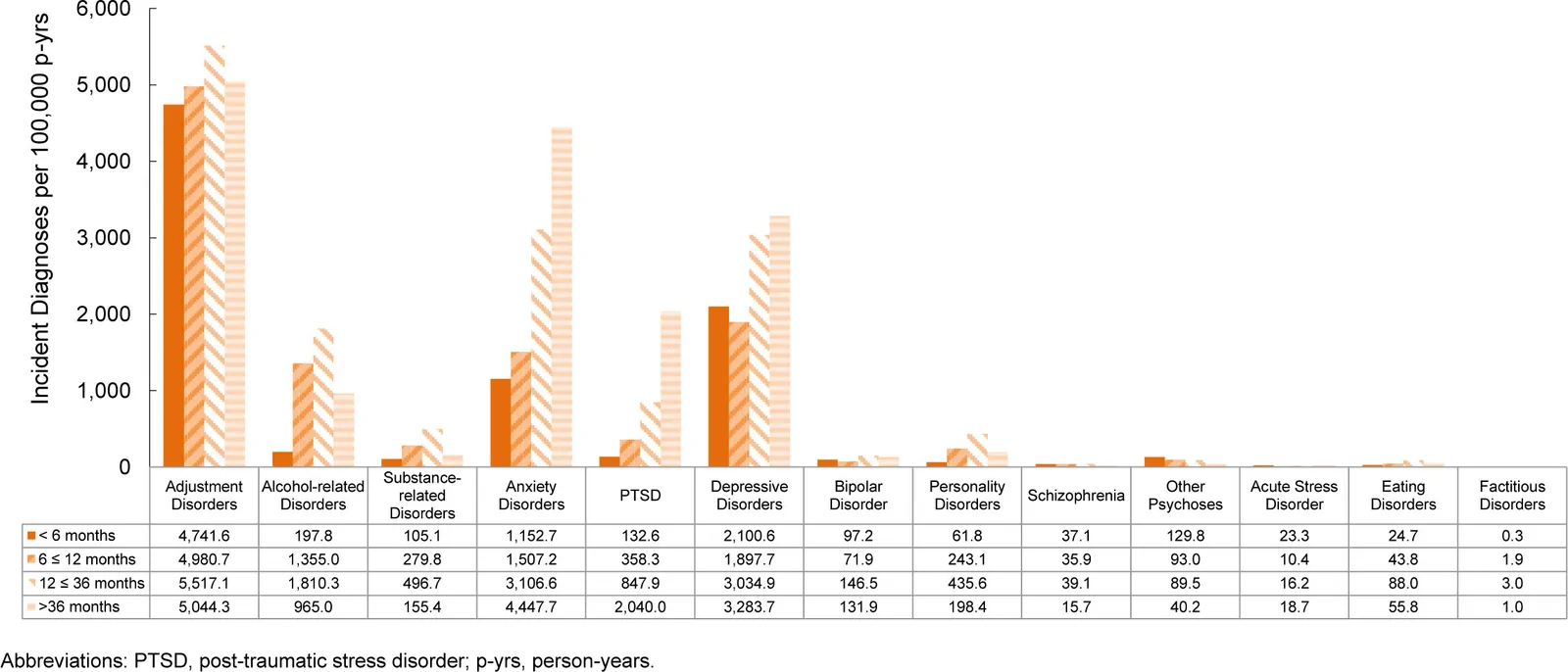
Incidence Rates of Mental Health Disorder Diagnoses by Category and Time in Service, Active Component, U.S. Armed Forces, 2021–2025

## Discussion

The findings from this 5-year surveillance period underscore a substantial and evolving mental health burden among active component service members of the U.S. Armed Forces. While the overall incidence of mental health diagnoses peaked in 2023 before demonstrating a slight decline through 2025, the volume of health care provision (over 1 million incident diagnoses affecting more than 582,000 service members) highlights the persistent operational impact of behavioral health conditions. Adjustment, anxiety, and depressive disorders overwhelmingly drove this burden, accounting for nearly two-thirds (66.5%) of all incident diagnoses.

Consistent with historical trends, female service members generally experienced higher rates for most mental health conditions, disproportionately bearing the burden of eating and personality disorders. Furthermore, female diagnosis rates for adjustment and anxiety disorders were double those of their male counterparts. Conversely, male service members exhibited higher IRs for alcohol- and substance-related disorders, as well as schizophrenia in the later years of the surveillance period. Occupational analysis further highlights specific vulnerabilities: health care personnel experienced the highest rates of adjustment, anxiety, depressive, and post-traumatic stress disorders, potentially reflecting the compounding, chronic stress inherent to military medicine. In contrast, combat-related roles were most closely associated with alcohol and substance-related disorders, pointing to different environmental stressors and possibly different behavioral coping mechanisms among specialties.

The manifestation of mental health disorders shifted significantly across a service member’s career lifecycle. Early-career personnel (younger than 20 years of age or with less than 6 months of service) were most vulnerable to acute stress and psychotic disorders, which may reflect the initial stressors of transitioning into the rigorous military environment. Mid-career members (ages 20–24 years or 12–36 months of service) saw peaks in substance-related, bipolar, and personality disorders. As time in service and age increased, the clinical presentation shifted heavily toward cumulative or chronic conditions, with personnel older than age 40 years experiencing the highest rates of PTSD, anxiety, and depressive disorders.

Finally, the high rate of diagnostic comorbidity presents a substantial clinical challenge. Nearly half (48.4%) of the affected surveillance population received diagnoses in more than 1 mental health category, with adjustment and depressive disorders frequently co-occurring with conditions such as substance-related and bipolar disorders. This complicated clinical picture suggests that isolated, single-disorder treatment approaches may be insufficient for this population.

There are several limitations to interpreting the results in this report. First, this report was compiled based on standardized administrative records that may not be reliable indicators of the true burden of mental health disorders among military service members. Second, this report may underestimate the incidence of mental health disorders if service members did not seek appropriate care or received care not documented routinely as ICD-9/ICD-10-coded diagnoses (e.g., from counseling or advocacy support centers, chaplains), or if mental health disorders were not diagnosed or reported on standardized records of care (e.g., from private practitioners), or if diagnoses were miscoded or incorrectly transcribed on centrally transmitted records. Conversely, some conditions may have been erroneously diagnosed or miscoded (e.g., screening visits) as mental health disorders, which could contribute to an over-estimation of the true burden of disease. Lastly, these analyses summarize the experiences of individuals while serving in an active component of the U.S. military and do not include mental health disorders or problems that affected members of reserve components or veterans of recent military service who received care outside the MHS.

The DOW policy framework actively promotes help-seeking by protecting patient privacy during mental health and voluntary substance misuse treatments. ^7^ This confidentiality is reinforced by the Brandon Act’s mandate for unquestioned, expedited command referrals. ^6^ Notably, the 2023 peak in overall incidence and high volume of anxiety and adjustment disorders coincides with the early implementation of these initiatives aimed at reducing stigma and removing barriers to care. As service members use these protected mechanisms, an initial rise in diagnosed incidence may represent an indicator of increased care provision rather than a confirmed expansion of disease burden.

Mental health is an integral component of military readiness and operational lethality. Consistent epidemiological surveillance is a proven mechanism for monitoring health care provision, clinical treatment, and timeliness of care. ^9^ Ultimately, the high rates of diagnostic comorbidity and occupational vulnerabilities documented in this report reveal the need for multidisciplinary clinical care capable of managing complex, concomitant behavioral health needs throughout the force.
