# Supplementary material for: Diagnoses of Mental Health Disorders Among U.S. Active Component Service Members, 2021–2025
Source: MSMR. 2026 Aug 20;33(8):3–14. (PMC13645440)
Supplement: Supplementary file 1 [file msmr-33-8-3-s01.pdf]

FIGURE 3 SUPPLEMENT. Incidence Rates of Mental Disorder Diagnoses by Age Group and Category, Active Component, U.S. Armed Forces, 2021–2025

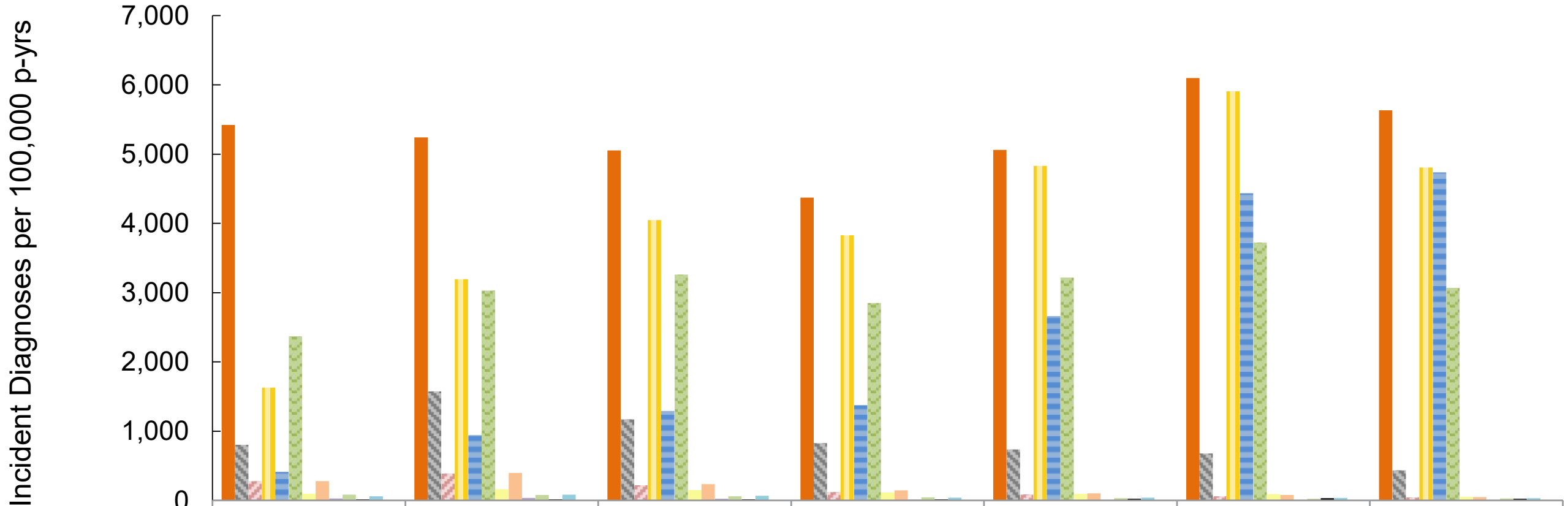

	<20	20–24	25–29	30–34	35–39	40–49	50+
Adjustment Disorders	5,418.8	5,240.5	5,051.7	4,373.3	5,060.7	6,097.8	5,633.5
Alcohol-related Disorders	802.9	1,575.7	1,171.1	824.6	736.7	677.9	436.9
Substance-related Disorders	279.2	387.8	220.3	120.9	85.1	59.1	42.0
Anxiety Disorders	1,630.2	3,193.5	4,049.0	3,828.3	4,829.5	5,907.7	4,804.9
PTSD	411.3	944.3	1,293.5	1,376.0	2,662.9	4,434.0	4,736.2
Depressive Disorders	2,368.7	3,031.4	3,262.3	2,848.7	3,216.1	3,722.6	3,067.4
Bipolar Disorder	94.2	161.3	150.0	112.7	93.0	92.1	52.5
Personality Disorders	278.8	395.9	236.0	145.8	102.3	78.8	48.7
Schizophrenia	26.9	34.1	26.0	13.9	8.7	6.5	9.2
Other Psychoses	84.3	80.5	57.7	43.5	30.1	25.1	27.5
Acute Stress Disorder	17.3	15.9	15.2	15.2	22.9	30.7	23.6
Eating Disorders	57.4	81.8	66.0	41.4	41.6	34.5	31.5
Factitious Disorders	1.6	1.8	1.6	1.4	0.7	0.3	2.6

Abbreviations: PTSD, post-traumatic stress disorder; p-yrs, person-years.

FIGURE 4 SUPPLEMENT. Incidence Rates of Mental Health Disorder Diagnoses by Branch of Service and Category, Active Component, U.S. Armed Forces, 2021–2025

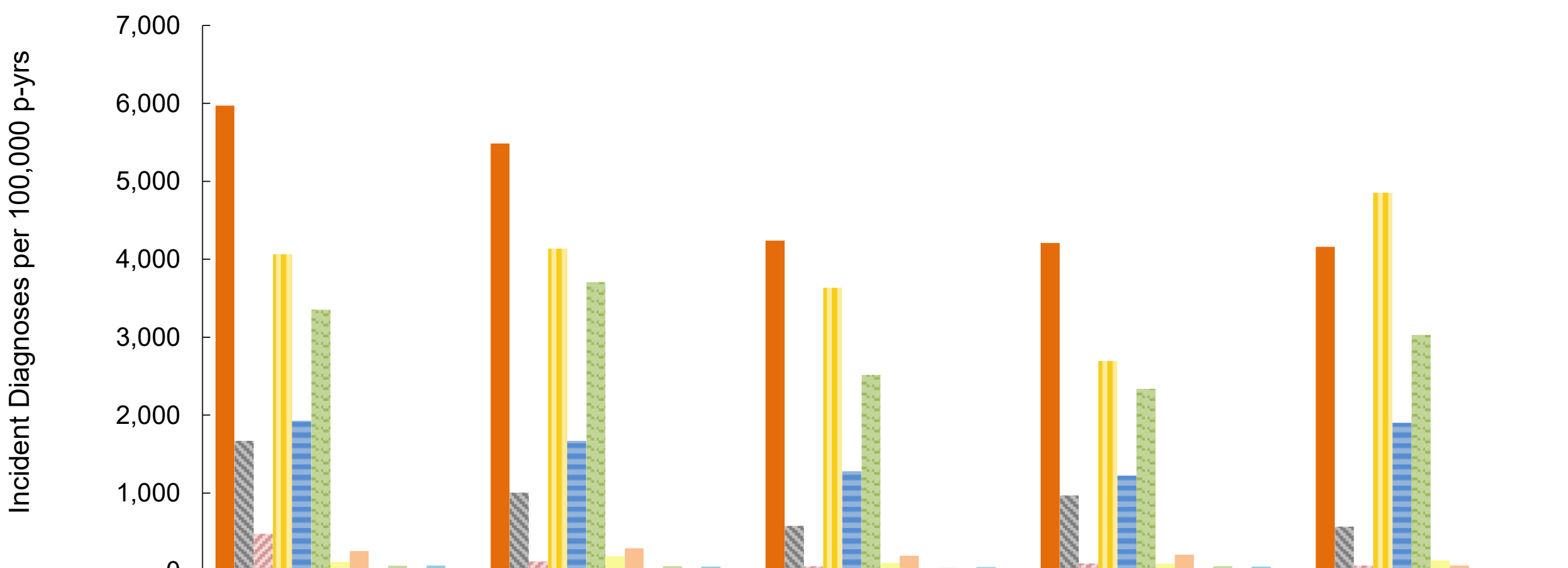

	Army	Navy	Air Force	Marine Corps	Coast Guard
Adjustment Disorders	5,969.7	5,486.1	4,238.1	4,209.7	4,159.3
Alcohol-related Disorders	1,672.3	1,006.8	580.0	968.6	569.0
Substance-related Disorders	478.1	123.4	61.8	97.4	72.2
Anxiety Disorders	4,062.6	4,135.2	3,634.6	2,696.4	4,852.8
PTSD	1,929.4	1,671.6	1,280.2	1,226.0	1,903.0
Depressive Disorders	3,352.2	3,708.6	2,517.8	2,338.5	3,028.8
Bipolar Disorder	118.3	188.7	103.9	94.7	136.9
Personality Disorders	257.5	290.9	195.9	210.8	72.2
Schizophrenia	25.9	22.3	17.6	23.5	15.3
Other Psychoses	68.3	59.4	41.1	59.4	32.7
Acute Stress Disorder	17.3	23.6	13.7	14.4	31.2
Eating Disorders	70.1	58.5	50.4	57.7	38.9
Factitious Disorders	2.7	0.9	0.8	0.7	0.0

Abbreviations: PTSD, post-traumatic stress disorder; p-yrs, person-years.

FIGURE 5 SUPPLEMENT. Incidence Rates of Mental Health Disorder Diagnoses by Military Occupation and Category, Active Component, U.S. Armed Forces, 2021–2025

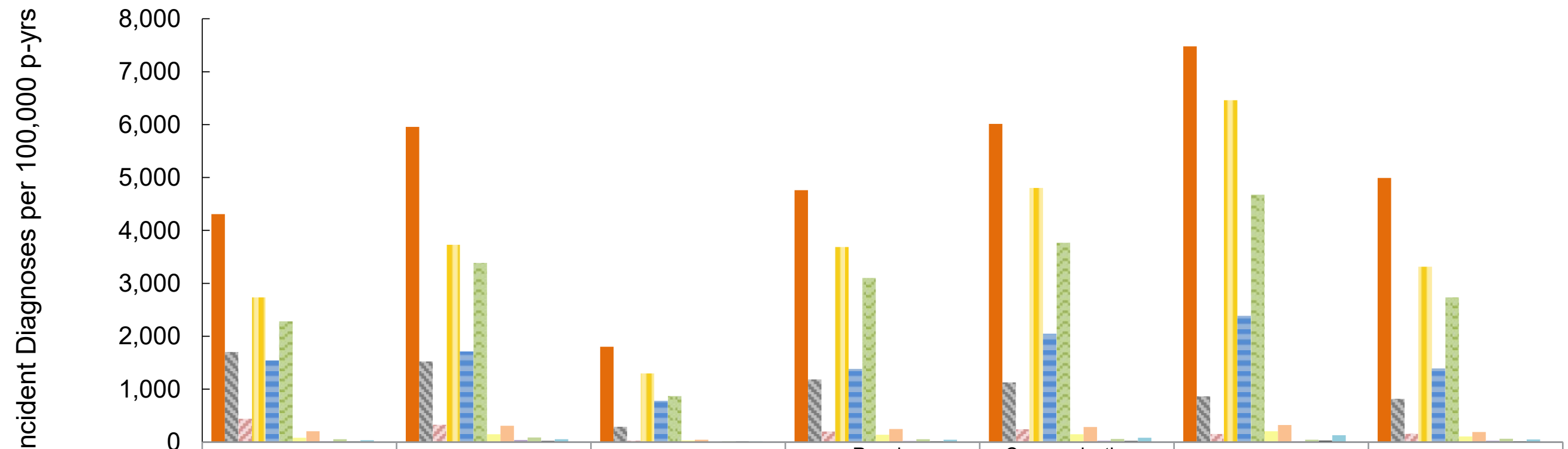

	Combat-related[a]	Motor Transport	Pilot, Air Crew	Repair, Engineering	Communications, Intelligence	Health Care	Other
Adjustment disorders	4,306.6	5,954.8	1,803.7	4,761.3	6,014.8	7,478.6	4,992.3
Alcohol-related disorders	1,705.3	1,523.6	289.2	1,187.4	1,130.3	867.0	816.0
Substance-related disorders	439.6	328.5	25.1	201.3	243.2	156.2	156.8
Anxiety disorders	2,733.0	3,727.5	1,298.5	3,687.2	4,801.9	6,460.0	3,312.5
PTSD	1,543.1	1,713.4	781.8	1,381.5	2,045.6	2,387.6	1,390.7
Depressive disorders	2,282.5	3,384.3	871.5	3,103.6	3,768.7	4,675.7	2,737.3
Bipolar disorder	83.7	148.5	30.4	141.3	150.4	207.6	108.1
Personality disorders	204.9	307.3	47.2	249.6	286.9	322.5	191.8
Schizophrenia	19.5	40.8	4.4	21.3	25.4	16.3	24.7
Other psychoses	54.9	86.4	15.9	53.1	60.8	44.6	66.9
Acute stress disorders	11.6	20.2	12.3	16.6	20.1	28.3	18.5
Eating disorders	36.2	56.8	12.8	45.9	83.3	130.3	51.6
Factitious disorders	2.0	0.5	0.0	1.3	1.7	1.0	1.5

Abbreviations: PTSD, post-traumatic stress disorder; p-yrs, person-years.
[a] Infantry, artillery, combat engineering.

FIGURE 6 SUPPLEMENT. Incidence Rates of Mental Health Disorder Diagnoses by Time in Service and Category, Active Component, U.S. Armed Forces, 2021–2025

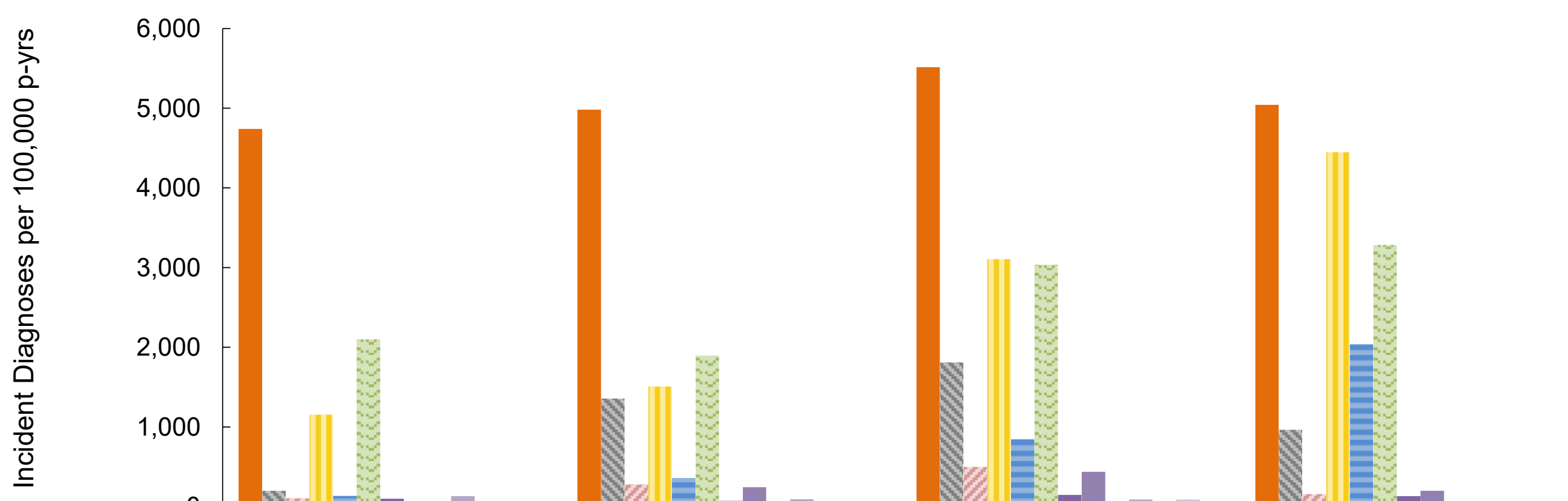

	< 6 months	6 ≤ 12 months	12 ≤ 36 months	>36 months
Adjustment Disorders	4,741.6	4,980.7	5,517.1	5,044.3
Alcohol-related Disorders	197.8	1,355.0	1,810.3	965.0
Substance-related Disorders	105.1	279.8	496.7	155.4
Anxiety Disorders	1,152.7	1,507.2	3,106.6	4,447.7
PTSD	132.6	358.3	847.9	2,040.0
Depressive Disorders	2,100.6	1,897.7	3,034.9	3,283.7
Bipolar Disorder	97.2	71.9	146.5	131.9
Personality Disorders	61.8	243.1	435.6	198.4
Schizophrenia	37.1	35.9	39.1	15.7
Other Psychoses	129.8	93.0	89.5	40.2
Acute Stress Disorder	23.3	10.4	16.2	18.7
Eating Disorders	24.7	43.8	88.0	55.8
Factitious Disorders	0.3	1.9	3.0	1.0

Abbreviations: PTSD, post-traumatic stress disorder; p-yrs, person-years.
